# Facile preparation and highly efficient sorption of magnetic composite graphene oxide/Fe_3_O_4_/GC for uranium removal

**DOI:** 10.1038/s41598-021-86768-0

**Published:** 2021-04-19

**Authors:** Aili Yang, Zhijun Wang, Yukuan Zhu

**Affiliations:** Institute of Materials, China Academy of Engineering Physics, P.O. Box 9071-7, Mianyang, 621907 China

**Keywords:** Environmental sciences, Pollution remediation

## Abstract

In this work, we reported for the first time a novel magnetic composite graphene oxide/Fe_3_O_4_/glucose-COOH (GO/Fe_3_O_4_/GC) that was facilely prepared from glucose through the hydrothermal carbonization and further combination with graphene oxide (GO). The chemical and structural properties of the samples were investigated. By the batch uranium adsorption experiments, the magnetic composite GO/Fe_3_O_4_/GC exhibits an excellent adsorption performance and fast solid–liquid separation for uranium from aqueous solution. GO/Fe_3_O_4_/GC (the maximum adsorption capacity (*Q*_m_) was 390.70 mg g^−1^) exhibited excellent adsorption capacity and higher removal rate (> 99%) for U(VI) than those of glucose-COOH (GC) and magnetic GC (MGC). The effect of the coexisting ions, such as Na^+^, K^+^, Mg^2+^, Ca^2+^, and Al^3+^, on the U(VI) removal efficiency of GO/Fe_3_O_4_/GC was examined. The equilibrium sorption and sorption rate for the as-prepared adsorbents well fit the Langmuir model and pseudo second-order kinetic model, respectively. The thermodynamic parameters (*ΔH*^0^ = 11.57 kJ mol^−1^ and *ΔG*^0^ < 0) for GO/Fe_3_O_4_/GC indicate that the sorption process of U(VI) was exothermic and spontaneous. Thus, this research provides a facile strategy for the preparation of the magnetic composite with low cost, high efficiency and fast separation for the U(VI) removal from aqueous solution.

Nowadays, carbonaceous materials, such as activated carbon^1^, carbon nanotubes^2^, carbon fibre^3^, and mesoporous carbon^4^, have been widely applied due to their availability, acid–base stability, and thermal resistance. These carbonaceous materials generally are fabricated via high temperature reaction^5^, pyrolysis^6–8^, gasfication^9^, electrospinning technique^10^, etc. Amongst these techniques, hydrothermal carbonization (HTC) has become a proficient synthesis technique owing to its cheapness, simplification, mild reaction conditions, and lack of any organic solvent and toxic waste. The fabrication of the biomass modification product by HTC process is one of the hot spots in recent years^11–13^. As a branch of carbonaceous materials the HTC materials from biomass have emerged since 1913^14^, and exhibited significant potential in various fields, such as adsorption^15–18^, catalysis^19^, fuel cell^20^, and energy storage/conversion^21–23^. Among the potential precursors for the preparation of HTC materials, glucose as a promising candidate has drawn much attention^24^. Glucose with low-cost and non-toxicity is a natural organic biomass, and reacts with heavy metals and influence their migration behaviour.

With increasing development of all kinds of industry, severe water pollution caused by reckless discharge into water has critically threatened human health and ecosystem^25^. Adsorption is a popular technique to resolve water contamination problems due to its low energy consumption, easiness, effectiveness, and no secondary pollution^26,27^. The glucose-based adsorbents have been proved to be promising for the removal of various pollutants, such as antibiotics^28^, organic pollutants^29^, gas pollutants^30^, dyes^31^, and heavy metal ions^32,33^. However, glucose treated solely by a hydrothermal approach possesses poor pore configuration, undeveloped porosity and low adsorption capacity, which make it being rarely applied in the removal of the pollutants^12^. Therefore, it is significant to develop novel glucose derivatives with numerous functional group in order to enhance the property.

Graphene oxide (GO) has been proved a popular candidate as a template for fabricating other functional nanomaterials, owing to its unique layered structure and remarkable physicochemical properties, such as high surface area, hydrophobicity, conductivity, and elasticity. Moreover, GO as an adsorbent can efficiently capture contaminants in water for water remediation^34^. However, the excellent dispersion of GO increases difficulty in separation between GO and treated solution, and tend to agglomerate after adsorption which lessens the adsorption capacity of GO^35^. To overcome these drawbacks of GO, the functionalization of GO with other materials, such as glucose, has become a promising tendency^36^. The addition of inexpensive glucose onto GO can effectively reduce the production cost of the adsorbent, and obtain abundant active oxygen group simultaneously. Xie et al.^37^ synthesized glucose-based carbon nanosheets by an integrated GO-confined nanospace directed KOH-activated process for the removal of sulfamethazine. Martín-Jimeno et al.^38^ prepared HTC xerogels via hydrothermal carbonization of glucose in the presence of GO as morphology directing agent and KOH activation method for CO_2_ and dye adsorption. However, the preparation methods for these glucose-based composites have some major drawbacks, such as high energy cost, operation complexity, and soli-liquid separation difficulty.

In order to overcome the separation problem, green and inexpensive magnetic nanoparticles, such as Fe_3_O_4_, have drawn considerable attention due to their environmentally friendliness and unique magnetic behavior^39–41^. The existence of Fe_3_O_4_ makes solid substance be rapidly separated from liquid phase only through an external magnetic field to shorten the wastewater treatment period. To our best knowledge, few researches focus on the preparation of the magnetic glucose-based adsorbent with GO as the template for the removal of uranium. Herein, in the present work, a magnetic GO-functionalized HTC adsorbent graphene oxide/Fe_3_O_4_/glucose-COOH (GO/Fe_3_O_4_/GC) was synthesized using glucose as an initial material via hydrothermal carbonization and magnetization reaction, which aims to develop a novel high-efficiency adsorbent for the removal of U(VI) from nuclear waste influent. The fabrication process and magnetization curve of GO/Fe_3_O_4_/GC (magnetization saturation value is 23.79 emu g^−1^) are shown in Fig. 1. The samples were characterized by elements analysis, crystal phase, functional group, and thermal stability. To evaluate the removal performance of the samples for U(VI), the batch adsorption experiments were carried out, and the kinetic and thermodynamic parameters in the adsorption process were provided.

**Figure 1:**
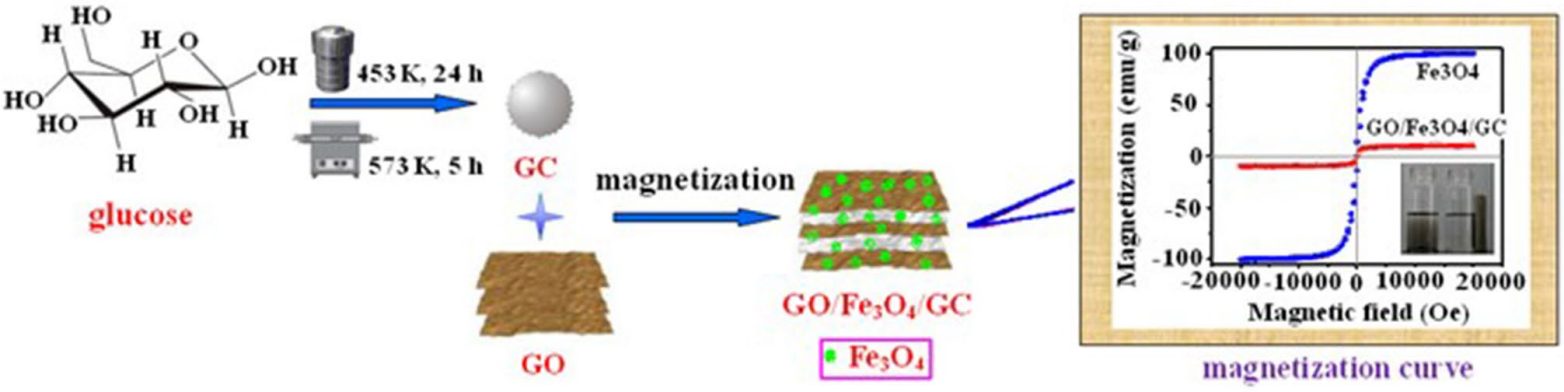
The fabrication process and magnetization curve of GO/Fe_3_O_4_/GC.

## Results and discussion

### Characterization

**Figure 2:**
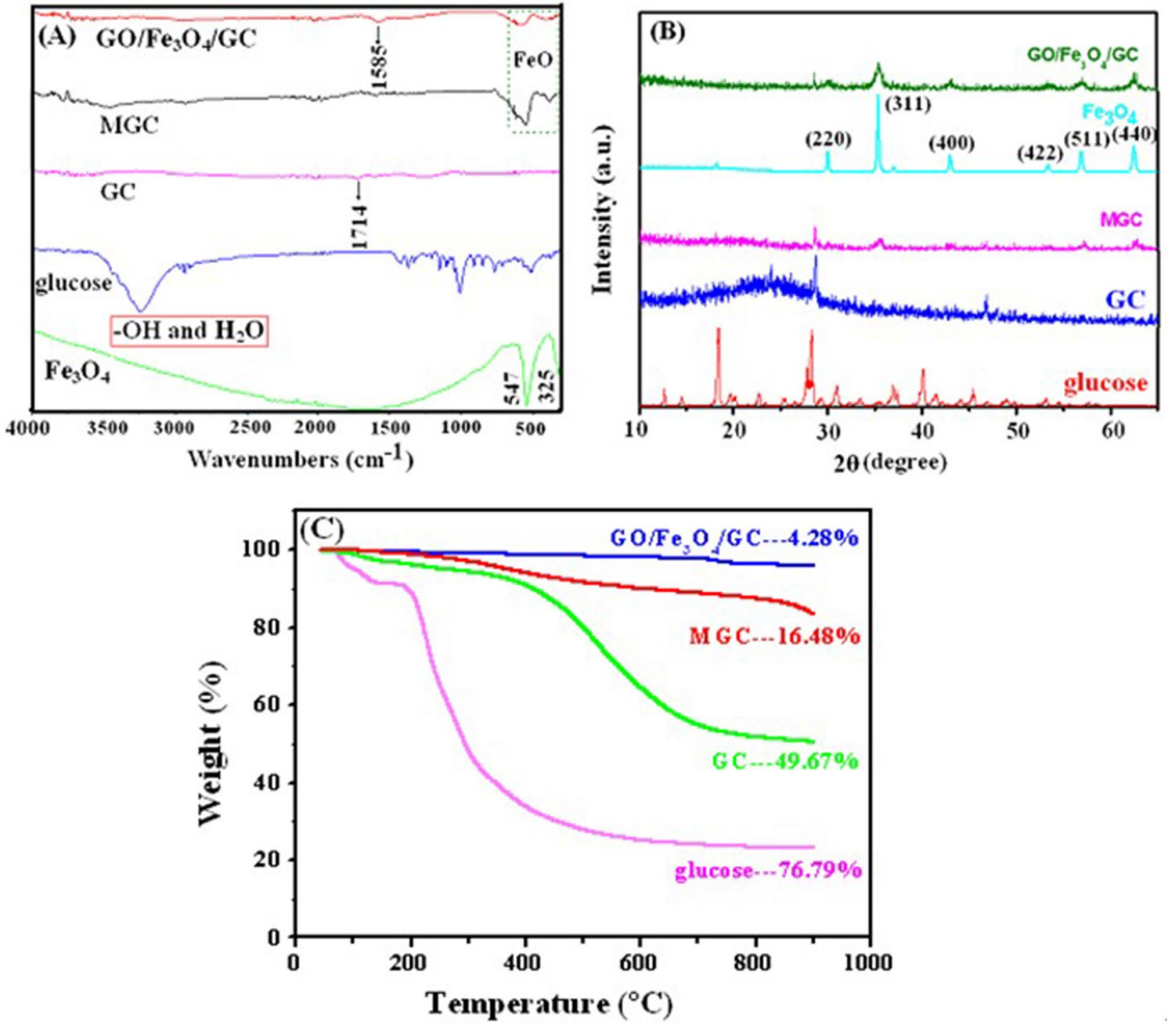
IR spectra (**A**), XRD patterns (**B**) and TGA curves (**C**) of the samples.

The Fourier transform infrared (FTIR) spectra of glucose, Fe_3_O_4_, GC, MGC and GO/Fe_3_O_4_/GC were shown in Fig. 2A. In the FTIR spectrum of GC, most of the characteristic peaks disappeared compared to glucose, but the intensity of the peak at 1714 cm^−1^ attributed to the group –COOH was higher than that of glucose, which indicated that GC was successfully obtained after hydrothermal and calcination treatment, and the number of carboxyl, carbonyl and ester groups significantly increased on the surface. These characteristic peaks of GC were similar to those of HTC-COOH reported in the reference^32^. In the FTIR spectrum of GO/Fe_3_O_4_/GC, the characteristic peaks at ~ 567 cm^−1^ and ~ 352 cm^−1^ belonged to Fe–O stretching vibration^42^ appeared, suggesting that the magnetic composite GO/Fe_3_O_4_/GC was successfully prepared.

The crystal phases of the samples were presented in Fig. 2B. In the X-ray diffraction (XRD) pattern of glucose, the strong diffraction peaks reflected great crystallization of glucose. However, the crystal phase of GC is amorphous owing to the calcination process in 300 ℃, which accords with the reference^43^. The structures of MGC and GO/Fe_3_O_4_/GC are also amorphous due to the existence of GC. Moreover, the characteristic peaks of the purchased Fe_3_O_4_ at 2θ = 29.94°, 35.30°, 42.98°, 53.38°, 56.84°, and 62.46°, corresponded to the (220), (311), (400), (422), (511), and (440) planes of magnetite Fe_3_O_4_ with a face-centered cubic structure (JCPDS No. 75-0033)^44^, were clearly shown in Fig. 2B. The XRD patterns of GO/Fe_3_O_4_/GC and MGC are basically consistent with those of Fe_3_O_4_, but the intensity of the peaks has significantly reduced because of the addition of GO and GC, revealing that the decoration process of Fe_3_O_4_ did not change the crystal phase of magnetite composite.

To reveal the thermal stability of the samples, the thermogravimetric analysis (TGA) curves of glucose, GC, MGC and GO/Fe_3_O_4_/GC in the range of temperatures from 30 to 900 °C are shown in Fig. 2C. Glucose is a kind of organic compound, and starts to decompose at 200 °C by TGA. GC is obtained from glucose through hydrothermal and carbonization at high temperature. While in the preparation process of GO/Fe_3_O_4_/GC and MGC inorganic substance Fe_3_O_4_ was introduced into their molecular. In general, thermal stability of organic substances is poorer than that of inorganic substances. In the structure of GO/Fe_3_O_4_/GC there are more interaction including electrostatic interaction, ionic interaction, and π-π stacking interaction than MGC. Therefore, the thermal stability of GO/Fe_3_O_4_/GC is higher than those of glucose, GC and MGC. The composite GO/Fe_3_O_4_/GC presented the smallest weight loss (4.28%) when the temperature was up to 900 °C, which showed that GO/Fe_3_O_4_/GC had the excellent thermal stability and almost no thermal decomposition took place. While the thermal stability of glucose, GC and MGC was poor, and the weight losses at 900 °C were 76.79%, 49.67% and 16.48%, respectively.

**Figure 3:**
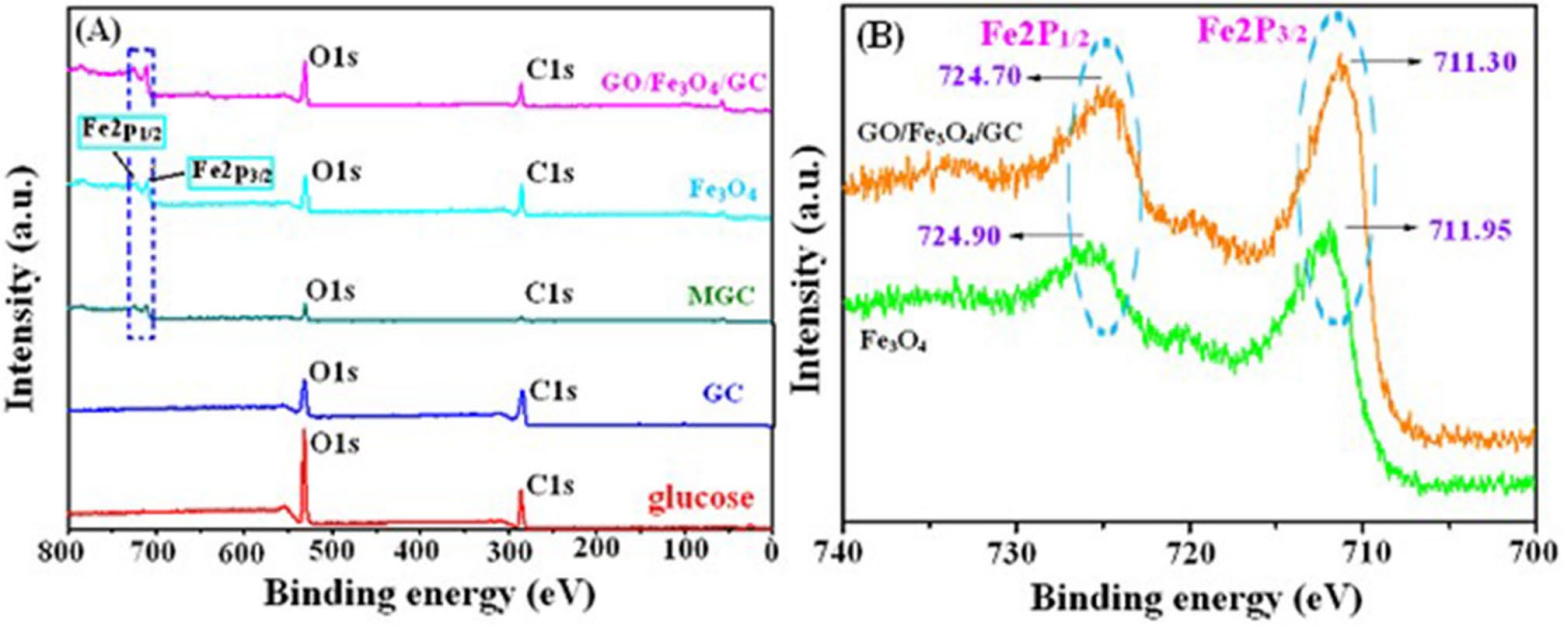
XPS survey spectra of Fe_3_O_4_, glucose, GC, MGC and GO/Fe_3_O_4_/GC (**A**) and the high-resolution Fe 2*p* spectra of Fe_3_O_4_ and GO/Fe_3_O_4_/GC (**B**).

The element composition of Fe_3_O_4_, glucose, GC, MGC and GO/Fe_3_O_4_/GC was investigated by X-ray photoelectron spectrometer (XPS). The XPS survey spectrum of GO/Fe_3_O_4_/GC (Fig. 3A) shows that the evident characteristic peaks at about 285, 554, 711 and 725 eV attributed to C1*s*, O1*s* and Fe2*p*, respectively. In the high-resolution spectrum of Fe 2*p* (Fig. 3B), the peaks of Fe 2*p*_3/2_ and Fe 2*p*_1/2_ are located at 711.30 eV and 724.70 eV, respectively, indicating the presence of Fe_3_O_4_ in the composite GO/Fe_3_O_4_/GC.

**Figure 4:**
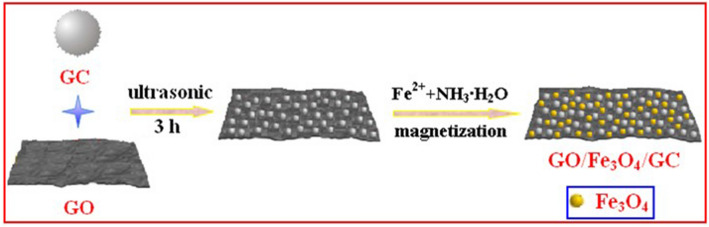
Schematic diagram of a possible formation mechanism of GO/Fe_3_O_4_/GC.

Based on the above characterization results, a possible formation mechanism for GO/Fe_3_O_4_/GC is illustrated in Fig. 4. Firstly, GO nanosheet is physically mixed with GC in an ultrasonic bath to form the complex GO · GC through hydrogen bond interaction as shown in Eq. (1). Then, Fe^n+^ (n = 2, 3) ions were formed in the above suspension by adding Fe^2+^ ions because a part of Fe^2+^ ions were oxidized to Fe^3+^ in air by the redox reaction as shown in Eq. (2). With the hydrolysis of Fe^3+^ and the addition of 30% ammonia solution the magnetic composite GO/Fe_3_O_4_/GC was obtained as expressed in Eqs. (3) and (4). Thus, both GC and Fe_3_O_4_ are immobilized on the surface of GO.

1 $${\text{GO}} + {\text{GC}} \to {\text{GO}} \cdot {\text{GC}}$$

2 $${\text{Fe}}^{2 + } {\mathop{\longrightarrow}\limits_{[{\text{O}}_{2} {\text{in air}}]}^{\text{redox reaction}}}{\text{Fe}}^{3 + } \to {\text{Fe}}^{n + } \;({\text{n}} = 2,3)$$

3 $${\text{Fe}}^{3 + } + {\text{H}}_{2} {\text{O}}\mathop{\longrightarrow}\limits^{{{\text{hydrolysis}}}}{\text{Fe}}({\text{OOH}})$$

4 $${\text{GO}} \cdot {\text{GC}}*{\text{Fe}}^{{{\text{n}} + }} \;({\text{n}} = 2,3) + {\text{Fe}}({\text{OOH}}) {\mathop{\longrightarrow}\limits_{{{\text{pH}} = 1}}^{{30\% \,{\text{NH}}_{3} \cdot {\text{H}}_{2} {\text{O}}}}}{\text{GO}}/{\text{Fe}}_{3} {\text{O}}_{4} /{\text{GC}}$$

### Adsorption tests

**Figure 5:**
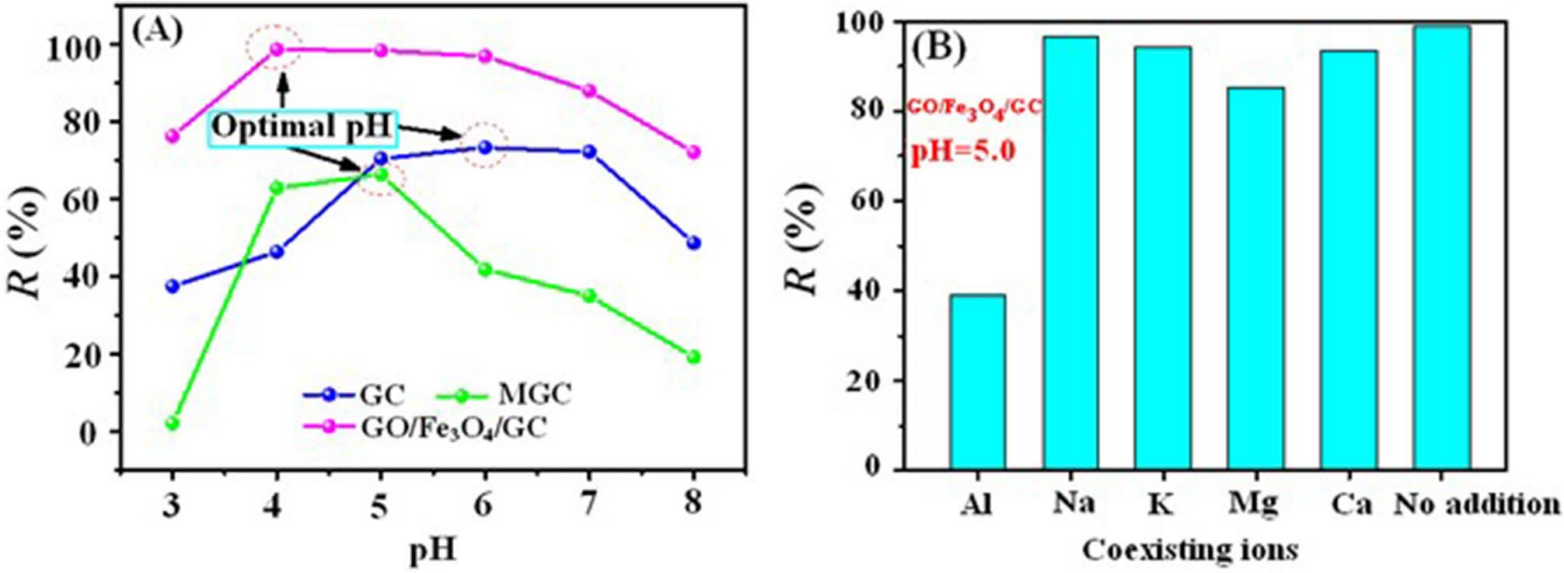
Effect of pH (**A**) and co-existing ions (**B**) on the U(VI) adsorption. *C*_(U)initial_ = 10 mg L^−1^, *C*_sorbent_ = 0.25 g L^−1^, *C*_co-existing ions_ = 2.5 g L^−1^, and contact time = 30 min.

The effect of the solution pH on the adsorption efficiency of the as-prepared adsorbents toward U(VI) is exhibited in Fig. 5A. The results show that the adsorption process for U(VI) obviously depends on the pH value of the solution. The pH value of the solutions greatly affects the surface charge of the samples. At pH < 4.0, the surface of the sorbents were protonated to form the positively charged surface, and then the electrostatic repulsion between these positive charge (including H_3_O^+^) and UO_2_^2+^ led to the poor adsorption capability for U(VI)^14^. With the appearance of the positive species (UO_2_(OH)^+^, (UO_2_)_3_(OH)_5_^+^, and (UO_2_)_4_(OH)_7_^+^) the removal efficiency of U(VI) significantly increased at pH 4.0–7.0 due to the electrostatic interaction between these complex uranium ions with positive charges and the negatively charged adsorbents. When pH is above 7.0, the negatively charged U(VI) species ((UO_2_)_3_(OH)_7_^−^ and UO_2_(OH)_3_^−^) are the dominant U(VI) species in solution which result in the reduction of the U(VI) removal efficiency^45^. The maximum removal rate for GC, MGC and GO/Fe_3_O_4_/GC was 66.30% (pH 6.0), 73.30% (pH 5.0), and 98.70% (pH 5.0), respectively. The sorption efficiency of U(VI) by GO/Fe_3_O_4_/GC was much higher than that of GC indicating that the addition of GO in the GC molecular enhanced greatly the adsorption property for U(VI). As a consequence, the optimal pH for GC, MGC and GO/Fe_3_O_4_/GC was selected as 6.0, 5.0 and 5.0 in the next U(VI) adsorption tests, respectively.

The influence of some important co-existing cations (e.g., Na^+^, K^+^, Ca^2+^, Mg^2+^, and Al^3+^) on U(VI) sorption by GO/Fe_3_O_4_/GC at 25 °C and pH 5.0 was shown in Fig. 5B. When no coexisting ions were added into the uranium solution, the removal rate of U reached 99.10%. It was clearly seen that Na^+^, K^+^, Mg^2+^ and Ca^2+^ had no significant competition effects on the sorption of U(VI). In contrast, the presence of Al^3+^ had a suppressive effect on U(VI) sorption. The results showed that the binding ability of cations to U(VI) followed the priority sequence: + 3 valence cations (e.g., Al^3+^) < + 2 valence cations (e.g., Mg^2+^ and Ca^2+^) < + 1 valence cations (e.g., Na^+^ and K^+^) which indicated that the better electrostatic interaction between high valence cations and the adsorbent GO/Fe_3_O_4_/GC result in the decrease of the adsorption efficiency for U(VI).

### Adsorption isotherm

**Figure 6:**
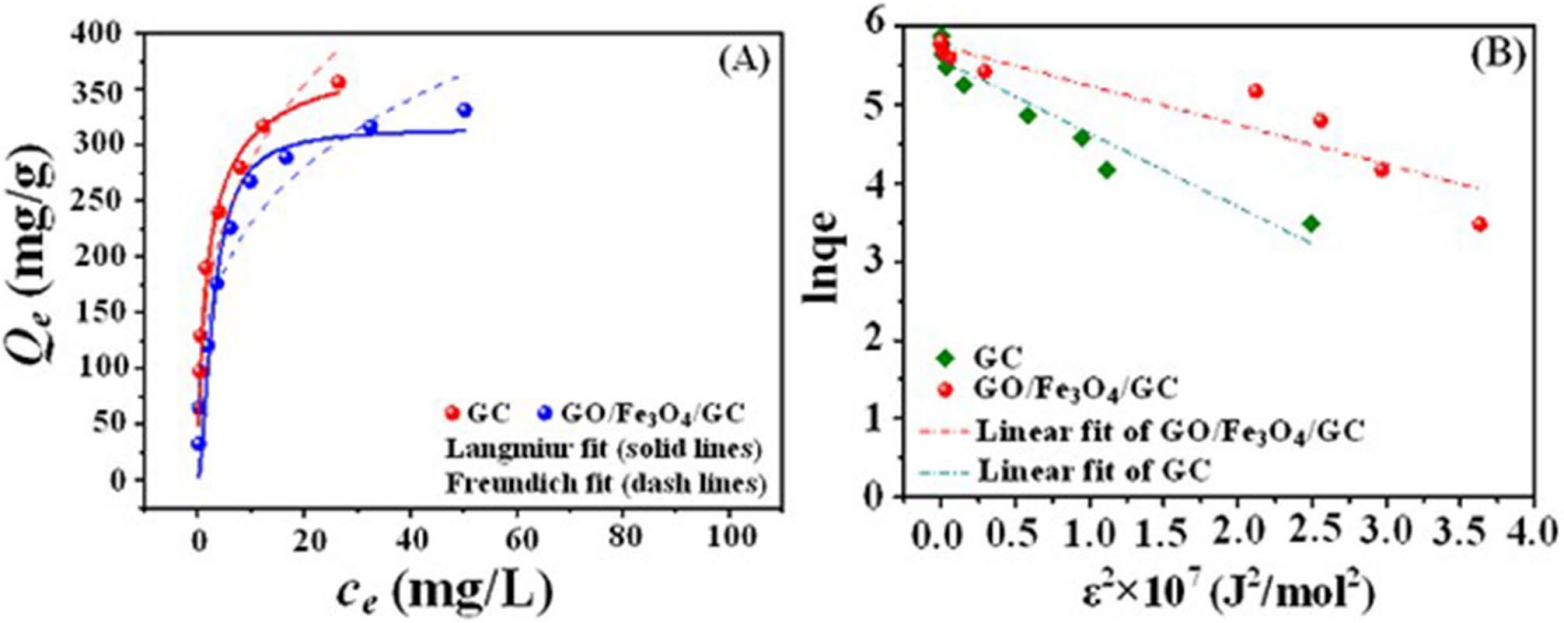
The fit results of Langmuir and Freundlich (**A**) and D–R (**B**) isotherm models for GC and GO/Fe_3_O_4_/GC. pH = 5.0 and 6.0, *C*_(U)initial_ = 5–150 mg L^−1^, *C*_sorbent_ = 0.15 g L^−1^, and contact time = 24 h.

The investigation of the adsorption isotherm reveals that how the adsorbate distribute between the liquid and the solid phase when the solution reach the adsorption equilibrium. The fit results of Langmuir, Freundlich and Dubinin–Radushkevich (D–R) isotherm models for the U(VI) adsorption on GC and GO/Fe_3_O_4_/GC are presented in Fig. 6. The Langmuir, Freundlich and D–R isotherm parameters are calculated and listed in Table 1. It was clearly seen that the Langmuir equation of the adsorbents fitted well the experimental data with a higher correlation coefficient compared to Freundlich and D–R adsorption isotherm models, implying that the adsorption of U(VI) onto the surface of GO/Fe_3_O_4_/GC is a monolayer coverage and the chelation behavior with functional groups of GO/Fe_3_O_4_/GC. The essential characteristic of the Langmuir isotherm are commonly expressed as the separation factor (*R*_L_) [Eq. (5)]^46^:

5 $$R_{L} = \frac{1}{{1 + k_{L} c_{0} }},$$

where *c*_0_ is the initial adsorbate concentration (mg L^−1^). The *R*_L_ value is related to the strength of the adsorption. The values of *R*_L_ > 1, *R*_L_ = 1, 0 < *R*_L_ < 1, and *R*_L_ = 0 indicate that weak, linear, strong or irreversible adsorptions, respectively. According to Table 1 it was seen that the *k*_L_ value of GO/Fe_3_O_4_/GC was 0.3420 and the calculated *R*_L_ value was 0.2262, indicating that strong adsorption between the adsorbent GO/Fe_3_O_4_/GC and U(VI).

**Table 1:** Parameters of Langmuir, Freundlich and D-R model for U(VI) adsorption on GC and GO/Fe_3_O_4_/GC.

Sorbents	Langmuir			Freundlich			D-R			
	*Q*_m_ (mg g^−1^)	*k*_L_ (L mg^−1^)	*R*^2^	*n*	*k*_F_ (mg^1−n^ L^n^ g^−1^)	*R*^2^	*Q*_m_ (mg g^−1^)	*β* (mol^2^(J^2^)^−1^)	*E* (kJ mol^−1^)	*R*^2^
GC	396.85	0.5796	0.9825	3.20	138.90	0.9322	312.85	0.94	0.73	0.9141
GO/Fe_3_O_4_/GC	390.70	0.3420	0.9873	3.49	118.85	0.9275	260.83	0.50	1.00	0.8646

According to Langmuir isotherm fit result, the maximum sorption capacity (*Q*_m_) of U(VI) on GC and GO/Fe_3_O_4_/GC was determined to be 396.85 mg g^−1^ and 390.70 mg g^−1^, respectively, higher than those of the previously reported glucose-based materials (see Table 2), which indicated that GO/Fe_3_O_4_/GC was a promising adsorbent for the treatment of the uranium-bearing wastewater. The fit for the data for the lowest uranyl concentrations is poor, which might result from the poor adsorption efficiency of the as-prepared adsorbent for the lower concentration uranium solutions. In this study, the as-prepared GO/Fe_3_O_4_/GC is a more promising adsorbent compared to other GO-based adsorbent due to the use of glucose with low-cost, environmental friendliness and anti-bacterial property as an initial material. The loading-U(VI) GO/Fe_3_O_4_/GC can be rapidly separated from the liquid phase through external magnetic fields due to the presence of magnetic Fe_3_O_4_.

**Table 2:** Comparison of *Q*_m_ of GO/Fe_3_O_4_/GC with reported other glucose-based sorbents for U(VI) adsorption.

Sorbents	pH	Contact time	m/V (g L^−1^)	*Q*_m_ (mg g^−1^)	References
HTC	6.0	50 min	0.2	62.7	^48^
HCSs–PO_4_-3	5.0	30 min	0.2	285.70	^49^
HTC–COOH	4.5	22 h	0.5	163	^32^
GC	6.0	24 h	0.15	396.85	This work
GO/Fe_3_O_4_/GC	5.0	30 min	0.15	390.70	This work

The Dubinin–Radushkevich (D–R) model is adopted to better explain the U(VI) adsorption behaviour (chemical adsorption or physical adsorption) onto the adsorbents. According to the D-R isotherm parameters, the obtained *E* values reveal the physical or chemical sorption mechanism. According to the literature^47^, if *E* lies between 8 and 16 kJ mol^−1^, the sorption process takes place chemically whereas *E* < 8 kJ mol^−1^ follows the physical sorption. For GC and GO/Fe_3_O_4_/GC, low *E* value (< 8 kJ mol^−1^) obtained in this study suggested that the adsorption process was mainly physical adsorption, which was in accordance with the reference^47^.

### Adsorption kinetics

**Figure 7:**
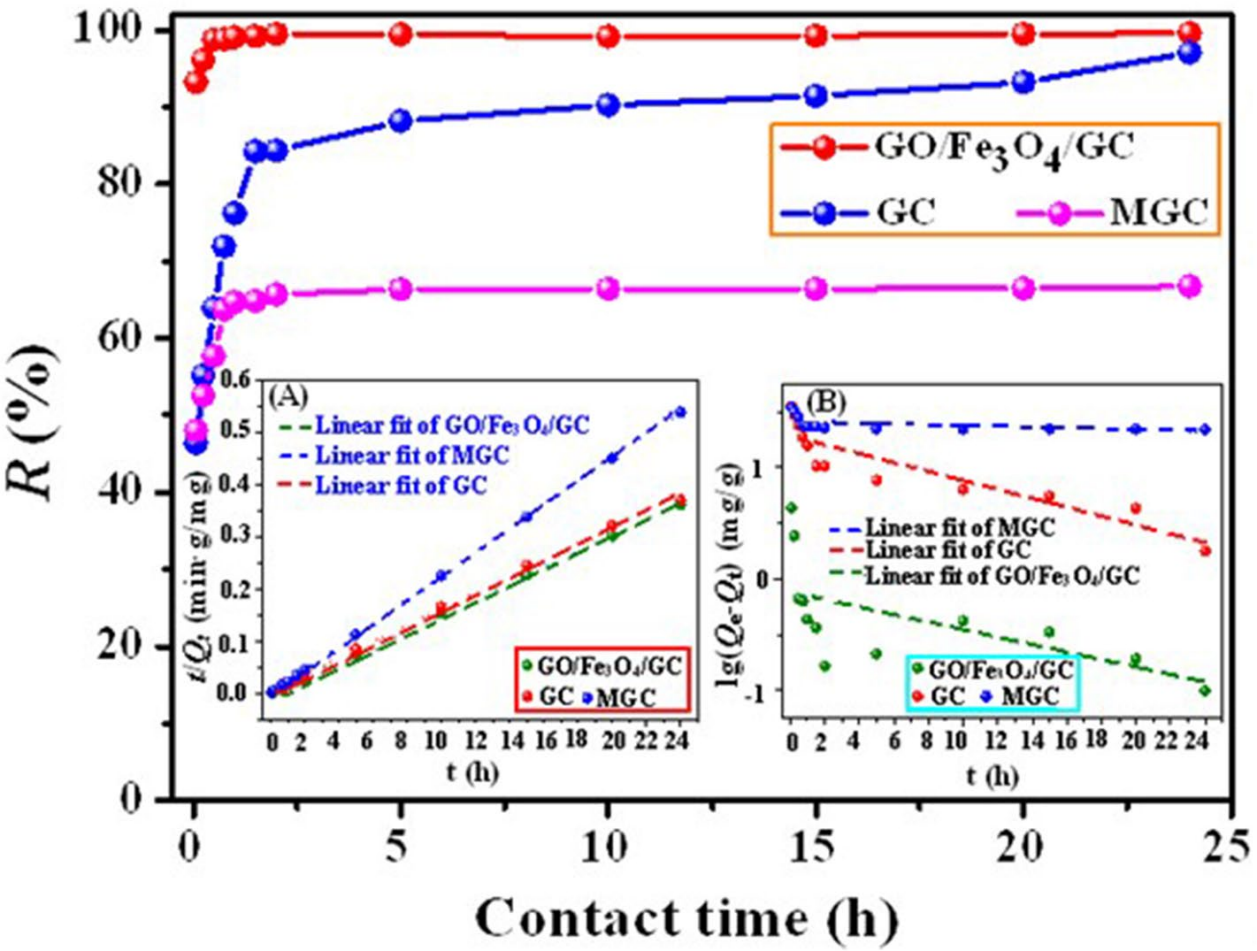
Influence of contact time on U(VI) sorption by GC, MGC and GO/Fe_3_O_4_/GC. Insert A and B are linear fit of the pseudo first-order and pseudo second-order kinetics models, respectively. pH = 5.0 and 6.0, *C*_(U)initial_ = 10 mg L^−1^, and *C*_adsorbent_ = 0.15 g L^−1^.

The adsorption kinetic mechanism is controlled by a mass transfer process involving equilibsrium time as well as physical and chemical adsorption characteristics. Figure 7 presents the time-dependent U(VI) adsorption rate over contact time ranging from 5 min to 24 h at initial U(VI) concentration of 10 mg L^−1^ by GC, MGC and GO/Fe_3_O_4_/GC. From Fig. 7 it is clear that the adsorption amount of U(VI) increases significantly with the extension of time until it reaches an equilibrium. As shown in Fig. 7, the adsorption kinetic of GO/Fe_3_O_4_/GC toward U(VI) indicated a fast adsorption process, and the remove of U(VI) could reach above 98% within 30 min. But the remove of U(VI) by GC could reach 97% after 24 h. Moreover, as presented in Fig. 7 (insert A and B), the correlation coefficients of pseudo second-order model were superior compared to pseudo first-order model which showed that the adsorption of UO_2_^2+^ ions onto GO/Fe_3_O_4_/GC was well fitted by the pseudo-second-order model. Adsorption kinetic parameters of the pseudo first-order and pseudo second-order model for GC, MGC and GO/Fe_3_O_4_/GC were given in Table 3. The results suggested that chemisorption is the rate-controlling step, implying the strong complexation between U(VI) ions and organic functional groups on the structures of GC, MGC and GO/Fe_3_O_4_/GC^50^.

**Table 3:** Parameters of the pseudo first-order and second-order kinetic models for U adsorption on GC, MGC and GO/Fe_3_O_4_/GC.

Sorbents	Pseudo first-order model			Pseudo second-order model		
	*Q*_*e*_ (mg g^−1^)	*k*_1_ (min^−1^)	*R*^2^	*Q*_*e*_ (mg g^−1^)	*k*_2_ *(*g (mg min)^−1^)	*R*^2^
GC	19.3598	0.0930	0.8016	63.82	0.0547	0.9989
MGC	26.2452	0.0098	0.2288	44.50	0.4763	0.9999
GO/Fe_3_O_4_/GC	0.7619	0.0788	0.3240	66.36	1.7671	1.0000

### Adsorption thermodynamics

**Figure 8:**
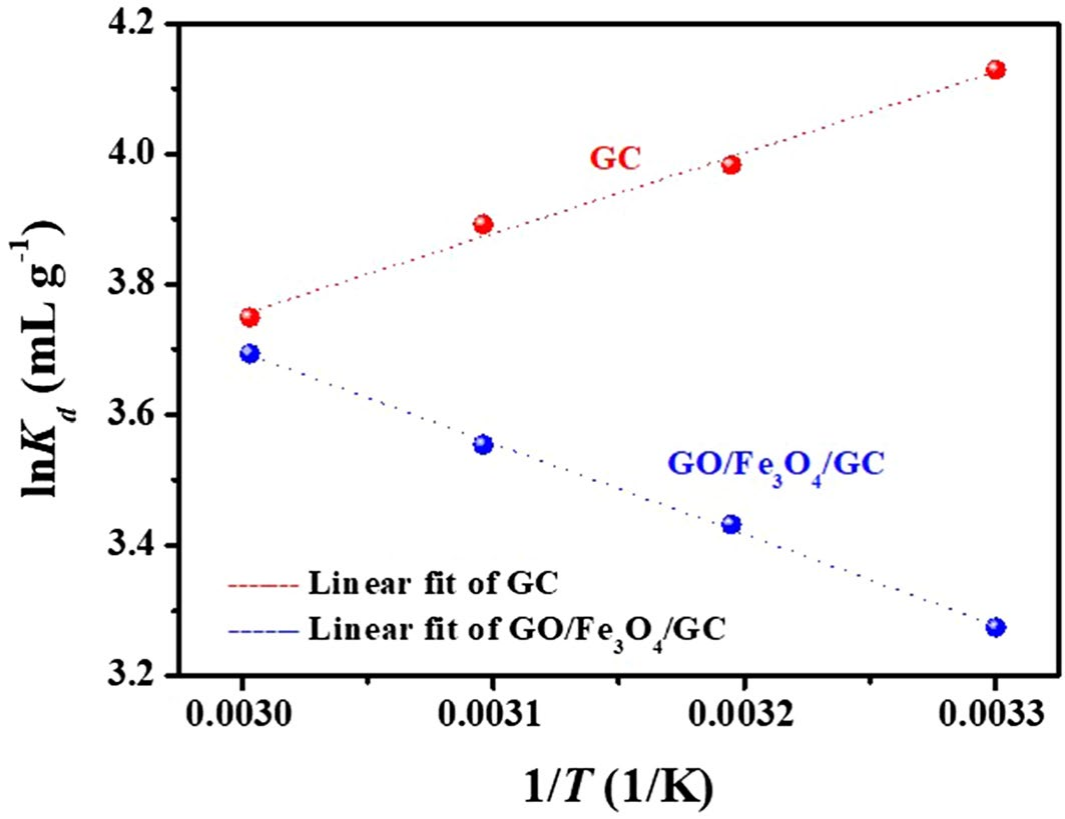
Plots of ln*K*_*d*_ versus 1/*T* for U(VI) adsorption onto GC and GO/Fe_3_O_4_/GC. pH = 5.0 and 6.0, *C*_(U)initial_ = 10 mg L^−1^, *C*_sorbent_ = 0.15 g L^−1^, *T* = 303 K, 313 K, 323 K and 333 K, and contact time = 24 h.

The plots of ln*K*_d_ versus 1/*T* onto GC and GO/Fe_3_O_4_/GC were shown in Fig. 8. The thermodynamic parameters such as enthalpy (Δ*H*^0^), entropy (Δ*S*^0^) and standard free energy (Δ*G*^0^) from 303 to 333 K in the adsorption processes were calculated according to Eqs. (6) and (7) and given in Table 4. The negative value of Δ*H*^0^ for GC reflected that the adsorption reaction was endothermic. While the positive value of Δ*H*^0^ for GO/Fe_3_O_4_/GC reflected that the adsorption reaction was endothermic. The positive Δ*S*^0^ and negative Δ*G*^0^ suggested that the spontaneity of the adsorption process.

6 $$\ln K_{d} = - \frac{{\Delta H^{0} }}{RT} + \frac{{\Delta S^{0} }}{R},$$

7 $$\Delta G^{0} = \Delta H^{0} - T\Delta S^{0} ,$$

**Table 4:** Thermodynamic parameters of U(VI) adsorption on GC and GO/Fe_3_O_4_/GC.

Sorbents	Δ*H*^0^ (kJ mol^−1^)	Δ*S*^0^ (J (mol k)^−1^)	Δ*G*^0^ (kJ mol^−1^)			
			303 K	313 K	323 K	333 K
GC	− 10.31	0.28	− 10.3948	− 10.3976	− 10.4004	− 10.4032
GO/Fe_3_O_4_/GC	11.57	65.42	− 8.2523	− 8.9065	− 9.5607	− 10.2149

### Regenerability of GO/Fe_3_O_4_/GC

Regeneration is an important aspect in the process of wastewater treatment in view of the cost saving. The reuse of GO/Fe_3_O_4_/GC was examined in this study. After the adsorption experiments, the obtained U-loaded GO/Fe_3_O_4_/GC was rinsed and washed with the regenerant (3 M HNO_3_) and the deionized water (DW) thoroughly until U(VI) ions were not detected in the rinse solution. Then, the dried and regenerated GO/Fe_3_O_4_/GC was reused for the further adsorption experiments (the adsorption conditions: pH = 5.0, *C*_0_(U) = 10 mg L^−1^, *T* = 25 ℃, adsorbent dosage = 0.15 g L^−1^, and contact time = 24 h). The results proved that GO/Fe_3_O_4_/GC was used repeatedly for the U(VI) adsorption, and the U(VI) removal rate reached 85.45% after five cycles.

## Materials and methods

### Materials

Uranyl nitrate (UO_2_(NO_3_)_2_·6H_2_O) was purchased from Xi’an Dingtian Chemical Reagent Co. (China). The stock solutions of uranium (5–150 mg L^−1^) were prepared by dissolving UO_2_(NO_3_)_2_·6H_2_O in DW and acidified with a small amount of concentrated HNO_3_. Glucose was obtained from Chengdu Keshi Reagent Co. (China). All reagents were of analytical grade and used without further purification. DW was used throughout the experiments.

### Synthesis of glucose-COOH (GC)

The hydrothermal carbon (HTC) was synthesized using glucose via a hydrothermal method. Briefly, 6 g of glucose were dissolved in 60 mL DW and placed in a Teflonlined autoclave at 180 ℃ for 24 h. After the autoclave was cooled to room temperature, the solid product HTC was filtered and washed with DW until the filtrate was colorless, and finally dried at 60 ℃ under a vacuum. Finally, carboxyl-rich glucose-COOH (GC) was obtained by heating HTC in a muffle for 5 h at 300 ℃.

### Synthesis of magnetic glucose-COOH (MGC)

Firstly, 0.5 g of GC was dissolved in 100 mL DW. Then, 30% NH_3_·H_2_O solution was added to the GC solution until the solution pH becomes 11. 1.25 g of FeSO_4_·7H_2_O was added slowly to the mixture under stirring. After stirring for 3 h, the black product (MGC) was collected by magnetic separation, and completely washed with DW and ethanol. Finally, MGC was dried at 50 ℃ in vacuum.

### Synthesis of magnetic composite GO/Fe_3_O_4_/GC

Firstly, GO was prepared from natural graphite by the modified Hummers method^51^. In a typical synthesis of GO/Fe_3_O_4_/GC, the mixture of 0.25 g of GO and 0.25 g of GC was dispersed in 100 mL DW under ultrasonic radiation for 3 h. Then, 30% NH_3_·H_2_O solution was added to the GO/GC solution until the solution pH becomes 11. 1.25 g of FeSO_4_·7H_2_O was added slowly to the mixture with continuous stirring. After stirring for 3 h, the magnetic black product (GO/Fe_3_O_4_/GC) was collected by magnetic separation, and washed with DW and ethanol. Finally, the product was dried at 50 ℃ in vacuum.

### Characterization

The FTIR spectra of the as-prepared adsorbents were obtained using a FTIR spectrometer (Bruker VERTEX 70, Germany). The crystal phases of the samples were characterized by the XRD pattern (Dandong 2700 model, China). The magnetic measurements of Fe_3_O_4_ and GO/Fe_3_O_4_/GC were conducted at 27℃ under a varying magnetic field (PPMS-9 ECII, USA Quantum Design Co.). XPS (Thermo Fisher ESCALAB 250, USA) was used to analyze the chemical composition of the samples. Thermal stability of the products was studied by a TGA system (Netzsch STA449F3, Germany) from 30 to 900 ℃ at a heating rate of 10 K min^−1^ under an argon flow.

### Adsorption experiments

The influence of pH, co-existing cations, contact time, initial U(VI) concentration, and temperature on the U(VI) removal was investigated. The U(VI) solution pH was adjusted to the desired value using HCl and NaOH. The as-prepared adsorbent was added to 20 mL solution and shaken in a shaker (Kangshi, China). After filtration, the U(VI) concentrations in solutions were determined by an MUA micro-quantity uranium analyzer (Beijing Yulun, China). The removal rate (*R*, %) and adsorption capacity (*Q*, mg g^−1^) were calculated according to Eqs. (8) and (9), respectively.

8 $$R(\% ) = \frac{{c_{0} - c_{t} }}{{c_{0} }} \times 100,$$

9 $$Q({\text{mg}}\,{\text{g}}^{ - 1} ) = \frac{{(c_{0} - c_{t} )}}{W} \times V,$$

where *c*_0_ (mg L^−1^) is the initial U(VI) concentration; *c*_*t*_ (mg L^−1^) is the U(VI) concentration at time *t*; *V* is the volume of the solution (L); *W* is the dosage of the adsorbent (g).

## Conclusions

In summary, three adsorbents GC, MGC and GO/Fe_3_O_4_/GC were facilely prepared using the inexpensive and environmentally benign glucose as a raw material for U(VI) capture via the simple hydrothermal carbonization and magnetization reaction. The optimum adsorption conditions for U(VI) with the initial concentration of 10 mg L^−1^ was at a pH of 5.0, a dosage of 0.15 g L^−1^, and contact time of within 30 min when using GO/Fe_3_O_4_/GC as the adsorbent. The existence of co-existing ions in solutions such as Na^+^, K^+^, Mg^2+^, Ca^2+^ and Al^3+^ had different influence on the removal of uranium by GO/Fe_3_O_4_/GC. Adsorption data of U(VI) by GO/Fe_3_O_4_/GC were in good agreement with Langmuir isotherm model and pseudo-second-order kinetic model. The composite GO/Fe_3_O_4_/GC exhibited excellent U(VI) sorption capacities (390.70 mg g^−1^), and a faster adsorption rate than those of GC, MGC and the previously reported glucose-based materials. The superior U(VI) uptake and fast solid–liquid separation after adsorption were mainly attributed to the abundant presence of GO and magnetic Fe_3_O_4_ particles in the molecule of GO/Fe_3_O_4_/GC. The facile production and high stability of GO/Fe_3_O_4_/GC reinforce its potential in the industrial purification of various pollutants, which paves the way for a new route to develop a novel glucose-based composite as a low-cost and highly efficient adsorbent to remove U(VI) from uranium-containing waste influents. The as-prepared GO/Fe_3_O_4_/GC has good regenerability which is very important in the practical application.
